# Effect of the Garlic Pill in comparison with Plavix on Platelet Aggregation and Bleeding Time

**Published:** 2012-09-22

**Authors:** H Fakhar, A Hashemi Tayer

**Affiliations:** 1 Department of Nursing, Arak University of Medical Sciences, Arak, Iran; 2 Department of Hematology, Arak University of Medical Sciences, Arak, Iran

**Keywords:** Bleeding Time, Garlic, Platelet Aggregation

## Abstract

**Introduction:**

Platelet aggregation plays a significant role in the etiology of cardiovascular diseases. Therefore, treatments to inhibit platelet aggregation can reduce the risk of coronary thrombosis. Several studies indicated that garlic can inhibit platelet aggregation. This study aimed to determine the effect of garlic in comparison with Plavix on platelet aggregation.

**Materials and Methods:**

In this randomized clinical trial, platelet aggregation and bleeding time was obtained from 36 healthy volunteers. Volunteers were randomly divided into 4 groups. The first, second and third groups respectively received 600, 1200 and 2400 mg garlic and the fourth group received 75 mg Plavix for three weeks. Afterwards, platelet aggregation and bleeding time were both evaluated and the results before and after the study were compared. Data were analyzed using SPSS software version 16.

**Results:**

Platelet aggregation induced by adenosine diphosphate and arachidonic acid agonists decreased in the groups that used 1200 or 2400 mg garlic. This difference was statistically significant (p<0.05). As compared to the other groups, the bleeding time also increased in those received 2400 mg garlic pill.

**Conclusion:**

Since garlic can inhibit platelet aggregation, it is suggested to use it as a supplementary treatment to reduce platelet aggregation is highly recommended.

## Introduction

Cardiovascular diseases are the important cause of death in the developed countries (1) and are related to various factors leading to increase in platelet aggregation. Since platelet aggregation is a major cause of heart diseases, using methods to prevent thrombosis and aggregation can prevent heart attacks (2). In fact, vessel wall injury is one of the major factors that cause platelet aggregation and thrombosis. Vascular endothelium produces platelet inhibitors, such as; nitric oxide, prostacyclin and adenosine diphosphatase (ADPase). However, when the vascular endothelium is damaged, the subendothelial compounds are exposed and cause platelet activation and aggregation. Therefore, treatments which inhibit platelet aggregation will reduce the risk of thrombosis and consequently prevent the cardiovascular diseases (3). Although, modern drugs can prevent from cardiovascular diseases but their use is limited due to their high cost, side effects and unexpected reactions (4). Regarding to these issues, herbal medicine is publicly accepted and its use is increasing frequently. Garlic is a herbal medicine which is known all over the world. During history, there has been a great deal of attention paid to the role of garlic in preventing heart diseases; but few scientific data exist to support medicinal and therapeutic features of garlic (5). The scientific name of the garlic is allium sativum and it is a member of bulbous plants, with its bulb having a sharp flavor, taste and smell (6). Some studies have been carried out about the effect of garlic on preventing the cardiovascular diseases and indicated that the consumption of garlic may prevents platelet aggregation and acts as an anti-thrombotic agent (7,9). Garlic is also used in different diets to accelerate wound healing and modulate the immune system as an anti-cancer, antibiotics and anti-oxidant agent (10).

In addition, treatment of metabolic, hyperlipidemia, hypertension and diabetes diseases (5,6). Therapeutic effects of garlic are mostly related to its organosulfur compounds such as cysteine sulfoxids and cysteines gamaglutamil. Other effective compounds found in garlic in large amount are diallyl disulfide (DADS), diallyl trisulfide (DATS) and methyl ajoene; all of which have anti-thrombotic effects (,). Experimental studies have proved that two substances, DADS and DAT, effectively inhibit platelets aggregation in presence of factors such as, oxidative agents and ADP (14). Also Garlic extracts can inhibit thromboxane formation and as a result prevent platelets from clumping together. Moreover, other non-sulfide compounds such as β-chlorogenin and quercetin which are extracted from this plant have been identified that also have inhibitory effects on platelet aggregation (15). Currently, few studies have been done on living creatures in this regard. This research aimed to investigate the effect of use of garlic on platelet aggregation inhibition and bleeding time. It also tries to compare these effects with plavix hat used by patients with heart disease.

## Materials and Methods

This randomized clinical trial study was conducted on 36 healthy adult male volunteers without having known digestive, coagulation and cardiovascular disorders in Arak university of medical science. The cases were selected through simple random sampling and consent form were read and signed by all volunteers. A blood sample (4.5 cc) was taken from every volunteer, and each sample was mixed with 0.5 cc sodium citrate 0.109 mM. The samples then were immediately sent to the laboratory and platelet aggregation test in presence of ADP, arachidonic acid, collagen and ristocetin agonists was performed on them. The test was done by an aggregometer machine; model PACKS-4 (Helena Lab, Beaumont, TX, USA). To measure the level of platelet aggregation, the blood samples were centrifuged by light rotation method for 10 minutes with 150-200 RPM in room temperature. Then the platelet rich plasma (PRP) was carefully gathered and directed to a siliconized tube. PRP could be kept in a room temperature up to 3 hours before the test. To obtain platelet poor plasma (PPP), the remaining blood was centrifuged by heavy rotation method for 20 minutes with 2000 RPM and then the supernatant was transferred to a siliconized tube. To standardize the platelet count, the number of platelets in each sample was counted using an automatic cell counter, the number was required to be in the range of 200 - 400 × 10^3^ per μL. In case that there are many platelets, the sample should be diluted using PPP. If there were fewer platelets in the sample, the control platelets in the Sphygmomanometer should be reduced to the same amount. Then the tube containing PPP was placed into the Sphygmomanometer and light pass was tuned to 100%. RPR was also kept at 37 °C for 2 minutes. Then four agonists including ADP, arachidonic acid, collagen and ristocetin were added; the amount was one tenth of the plasma volume. In this state, light absorption begins to change. This change takes about 3 minutes until the curve of the Sphygmomanometer gets straight. Light absorption reduction rate corresponds to the platelet aggregation rate and was calculated by the Sphygmomanometer. Bleeding time test (BT) was also performed on volunteers through Template method. In this test, a disposable device which equipped with blade was used. This makes vertical incisions in the epidermis. At first, the cuff of sphygmomanometer was wrapped around the arm. After inflating, the blood pressure was adjusted to 40 mmHg. Then three standard incisions are made in the inner surface of the forearm and a chronometer was turned on immediately. Every 30 seconds, the blood drop from the incision was absorbed by filter paper. When the filter paper was not colored by blood, the chronometer was stopped. The average time of three incisions was calculated and considered as BT. In the second stage, volunteers were randomly divided into four equal groups. The first, second and third group, respectively, received 600, 1200 and 2400 mg of garlic pill in three divided doses on a daily basis, and the fourth group received 75 mg Plavix (clopidogrel) on a daily basis for 3 weeks. Each group had a representative, who was responsible for controlling the samples about any probable side effects and also for avoiding any possible errors in taking drugs. All the samples were investigated about the exit factors every day. Following three weeks giving the last dose, the blood samples of all the volunteers were taken to determine platelet aggregation. BT test was also done in this stage.

**Statistical Analysis**

Collected data was analyzed using paired and two independent sample t-test with SPSS software version 16 and a P_value less than 0.05 was considered being statistically significant. 

## Results

All the cases were male, aged 19-24 yrs with mean age of 20.3±1.6 yrs and weighed 56-78 kg with mean weight of 67.2±4.2 kg. The findings showed that there was no significant difference between platelet aggregations resulted from ADP, arachidonic acid and collagen agonists before and after 3 weeks of using 600 mg garlic pill every day. As shown in Table I, this difference in ristocetin activity levels in inducing platelet aggregation was significant (P=0.03). Table I showed that there was no significant difference between the mean of arachidonic acid and collagen activity percentage before and after 3 weeks of daily consumption of 1200 mg garlic pill, but the difference in the activity of ADP (P=0.03) and ristocetin (P=0.01) was significant. In the third group, the obtained results proved that there was a significant difference between the platelet aggregation level from utilized agonists before and after 3 weeks of daily consumption of 2400 mg garlic pill. The results in the fourth group showed that there was a meaningful difference between the mean percentage of ADP (P=0.001), collagen (0.01) and arachidonic acid activities (P=0.001) before and after 3 weeks of daily consumption of plavix. However, this difference was not significant for ristocetin activity (P=0.4). Arachidonic acid activity level in inducing platelet aggregation was significantly reduced in this group after garlic consumption (Table I). The results of BT indicated that although it is in normal range (1– 9 min) in all groups, there was a significant difference between before and after use of garlic in third group compared to first and second groups (Table II).

**Table I:** Mean and standard deviation of agonists activity level on platelet aggregation in volunteers before and after using drug

Variable	Activity level of agonist (%)	Group 1 (600 mg garlic)	Group 2 (1200mg garlic)	Group 3 (2400mg garlic)	Group 4 (75 mg plavix)
ADP	Before After P- value	69.6 ± 9.92 60.75± 10.93 0.932	57.7 ± 6.28 36.7 ± 10.93 0.03	58.7 ± 15.2 44.6 ± 8.5 <0.001	65.4 ± 2.3 31.2 ± 3.4 <0.001
Arachidonic Acid	Before After P- value	62.9 ± 6.81 54.5 ± 4.26 0.826	61.7 ±15.1 43.39 ±13.2 0.1	68.2 ±10.7 48.1 ±9.1 0.007	68.3 ±5.5 32.6 ±2.45 <0.001
Collagen	Before After P- value	59.4 ±9.54 53.8 ± 10.81 0.1	58.7 ± 9.47 55.3 ± 10.43 0.1	59.7 ± 16.2 48.1 ± 19.1 0.01	59.9 ± 2.3 50.7 ± 9.1 0.01
Ristocetin	Before After P- value	65.8 ± 9.36 58.8 ± 4.7 0.03	65.1 ± 13.8 57.6 ± 11.6 0.01	57.3 ± 13.9 40.1 ± 12.3 0.006	70.3 ± 12.5 68.8 ± 17.4 0.4

**Table II:** Comparison of mean of bleeding time per minute in volunteers before and after using drug

**Bleeding time** **(per minute)**	**Before **	**After **	**p -value**
600 mg garlic	3.03± 0.352	4.75 ± 0.28	0.084
1200 mg garlic	2.08 ±0.43	5.34 ±0.436	0.08
2400 mg garlic	3.25 ± 0.456	8.28 ± 0.312	0.09
75 mg plavix	2.35 ± 0.52	6.7 ± 0.67	0.075

## Discussion

The present study indicated that there was not a significant difference in platelet aggregation with ADP, arachidonic acid, and collagen agonists before and after 3 weeks of daily use of 600 mg garlic pill. However, the difference was slightly meaningful for ristocetin. The use of garlic, only decreased platelet aggregation induced by ADP and ristocetin in the group that used 1200 mg dose of garlic pill, but it had no effect on collagen and arachidonic acid agonists. There was a meaningful difference between the before and after received of 2400 mg dose of garlic pill; in a way that the platelet aggregation induced by ADP, collagen, arachidonic acid and ristocetin agonists significantly decreased. The obtained results correspond with the previous studies indicating that the inhibitory effect of garlic on platelet aggregation in vitro and in vivo (,). Platelet aggregation plays an important role in the etiology of cardiovascular diseases (17). The conducted epidemiologic studies showed an inverse correlation between garlic consumption and the progression of cardiovascular diseases (15,18). Garlic prevents the cardiovascular diseases via some mechanisms. One of these mechanisms is inhibition of platelet aggregation. In vitro studies indicate that garlic prevents inhibition of platelet aggregation by inhibiting cyclooxygenase activity and thus thromboxane A_2 _(TXA_2_) and B_2 _(TXB_2_) formation, by suppressing mobilization of intraplatelet Ca2+, and by increasing levels of cyclic adenosine monophosphate (cAMP) and cyclic guanosine monophosphate (cGMP). Garlic also display strong antioxidant properties and activates nitric oxide synthase (NOS), leading to an increase in platelet-derived NO (17). Calcium (Ca2+) mobilization plays an important role in various aspects of platelet activation such as aggregation, shape change, and secretion. Stimulation of human platelets with various agonists elevates Ca2+ in 2 ways, i.e., the release of Ca2+ from intracellular stores that exist in dense granules, or by Ca2+ entry through platelet plasma membrane channels (19). It can also interact directly with the GPIIb/IIIa receptors, thus reducing the ability of platelets to bind to fibrinogen (17). Gillian et al, indicated that garlic can prevent calcium mobilization and platelet aggregation induced by both ADP and calcium ionophore A23187 (20). in other study, reported on the existence of adenosine deaminase and cAMP phosphodiesterase inhibitors as anticoagulant agents in garlic (21). These agents inhibit the ADP pathway and this mechanism is comparable to the function of plavix as an anti-platelet aggregation drug (22). The current study also investigated the effect of anti-platelet drug, Plavix, on platelet aggregation before and after its use. The results indicated that there was a significant difference in platelet aggregation induced by ADP, arachidonic acid, and collagen before and after 3 weeks of 75 mg Plavix every day. However, the difference was not significant in ristocetin. Plavix, in fact, inhibits platelet aggregation through selective and irreversible inhibition of the ADP interaction to its receptor on platelets. ADP interact with two different purinergic receptors on platelets, known as P2Y1 and P2Y12 Since 1997, plavix has been widely used to prevent and treat vascular thromboembolic diseases such as heart failure, stroke, and peripheral vascular diseases (23). This bind to P2Y12 receptors irreversibly, rendering the receptor unable to respond to ADP, thus reducing platelet fuction.This study also sought to find out the effect of different doses of garlic pill on BT coagulation test through template method. Although its level was in the normal range in all groups, there was a meaningful difference between BT in the group which received 2400 mg garlic pill and the groups which had 600 and 1200 mg of it. The result corresponds with other conducted studies (24). Ajoene compound which is found in garlic, affects the course of BT (24). It acts as a strong anti-platelet agent and reversibly inhibits platelet aggregation induced by arachidonic acid, adrenalin, collagen, and ADP. It also inhibits calcium ionophore A23187 in vitro. These findings are similar to some other research results about platelet activity inhibition by garlic (20,24). In addition, garlic increased partial thromboplastin time (PTT), thrombin time (TT), clotting time (CT). Similarly, it increases the activity of coagulation inhibitors such as anti-thrombin and protein C (25). fibrinolysis is also enhanced by garlic, resulting in dissolution of thrombi and clots in blood vessels (9). The effect of garlic on PTT and TT tests may be due to the inhibition of coagulation factors involved in the intrinsic and common clotting pathway such as 10, 9, 8, 5, 2 and 1. This study lacked facilities to evaluate the interaction between vascular endothelial cells and platelets after garlic consumption. It is suggested that this will be investigated in further studies. 

## Conclusion

In summary, considering the results obtained from this study and the others related to the anti-clotting nature of garlic in inhibiting platelet aggregation and according to various sources, the use of garlic is recommended to everyone who has no problem of taking it. Garlic can play an effective role in preventing and treating cardiovascular diseases. 

## References

[1] Rajadurai M, Stanely Mainzen Prince P (2007). Preventive effect of naringin on cardiac markers, electrocardiographic patterns and lysosomal hydrolases in normal and isoproterenol-induced myocardial infarction in Wistar rats. Toxicology.

[2] Wang SB, Tian S, Yang F, Yang HG, Yang XY, Du GH (2009). Cardioprotective effect of salvianolic acid A on isoproterenol-induced myocardial infarction in rats. Eur J Pharmacol.

[3] Hubbard GP, Wolffram S, de Vos R, Bovy A, Gibbins JM, Lovegrove JA (2006). Ingestion of onion soup high in quercetin inhibits platelet aggregation and essential components of the collagen-stimulated platelet activation pathway in man: a pilot study. Br J Nutr.

[4] Thippeswamy BS, Thakker SP, Tubachi S, Kalyani GA, Netra MK, Patil U (2009). Cardioprotective Effect of Cucumis trigonus Roxb on Isoproterenol-Induced Myocardial Infarction in Rat. American Journal of Pharmacology and Toxicology.

[5] Rahman K (2003). Garlic and aging: new insights into an old remedy. Ageing Res Rev.

[6] Karim A, Nouman M, Munir S, Sattar S (2011). Pharmacology and phytochemistry of pakistani herbs and herbal drugs used for tretement of diabetes. Int J Pharmacol.

[7] Borek C (2006). Garlic reduces dementia and heart-disease risk. J Nutr.

[8] Allison GL, Lowe GM, Rahman K (2006). Aged garlic extract and its constituents inhibit platelet aggregation through multiple mechanisms. J Nutr.

[9] Rahman K, Lowe GM (2006). Garlic and cardiovascular disease: a critical review. JNutr.

[10] Jafari RA, Ghorbanpoor M, SH D (2009). Study on immunomodulary activity of dieitary garlic in chickens vaccinated against avian influenza virus (subtype H9N2). Int J Poult.

[11] Butt MS, Sultan MT, Butt MS, Iqbal J (2009). Garlic: nature's protection against physiological threats. Crit Rev Food Sci Nutr.

[12] Boos CJ, Lip GY (2006). Blood clotting, inflammation, and thrombosis in cardiovascular events: perspectives. Front Biosci..

[13] Omar SH, NA (2010). A-w. A-w.Organosulfur compounds and possible mechanism of garlic in cancer.

[14] Chan KC, Hsu CC, Yin MC (2002). Protective effect of three diallyl sulphides against glucose-induced erythrocyte and platelet oxidation, and ADP-induced platelet aggregation. Thromb Res.

[15] Corzo-Martinez M, Corzo N, Villamiel M (2007). Biological properties of onions and garlic. Trends in food science & technology.

[16] Sobenin IA, Pryanishnikov VV, Kunnova LM, Rabinovich YA, Martirosyan DM, Orekhov AN (2010). The effects of time-released garlic powder tablets on multifunctional cardiovascular risk in patients with coronary artery disease. Lipids Health Dis.

[17] Rahman K (2007). Effects of garlic on platelet biochemistry and physiology. Mol Nutr Food Res.

[18] Ginter E, Simko V (2010). Garlic (Allium sativum L.) and cardiovascular diseases. Bratisl Lek Listy.

[19] Park WH, Kim HK, Nam KS, Shon YH, Jeon BH, Moon SK (2004). Inhibitory effect of GBH on platelet aggregation through inhibition of intracellular Ca2+ mobilization in activated human platelets. Life Sci.

[20] Allison GL, Lowe GM, Rahman K (2006). Aged garlic extract may inhibit aggregation in human platelets by suppressing calcium mobilization. J Nutr.

[21] Yeganeh M, Khojir Yeganeh Rad R (2007). The effect of garlic on coagulation tests. J Mazandaran Univ Med Sci.

[22] Hiyasat B, Sabha D, Grotzinger K, Kempfert J, Rauwald JW, Mohr FW (2009). Antiplatelet activity of Allium ursinum and Allium sativum. Pharmacology.

[23] Yanushi DW, Stan H (2011). Anti-platelet therapy: ADP receptor antagonists. British Journal of Clinical Pharmacology.

[24] Srivastava KC, Tyagi OD (1993). Effects of a garlic-derived principle (ajoene) on aggregation and arachidonic acid metabolism in human blood platelets. Prostaglandins Leukot Essent Fatty Acids.

[25] Chan KC, Yin MC, Chao WJ (2007). Effect of diallyl trisulfide-rich garlic oil on blood coagulation and plasma activity of anticoagulation factors in rats. Food Chem Toxicol.

